# Modified Supine Percutaneous Nephrolithotomy in the Elderly: Outcomes and Safety

**DOI:** 10.3390/jcm12144807

**Published:** 2023-07-21

**Authors:** Kylie Yen-Yi Lim, Amos Nepacina Liew, Zihui Ling, Weranja Ranasinghe, Philip McCahy

**Affiliations:** 1 Department of Urology, Monash Health, Melbourne, VIC 3168, Australia; 2 School of Medicine, Monash University, Clayton, VIC 3800, Australia; 3 School of Clinical Sciences, Monash University, Clayton, VIC 3800, Australia

**Keywords:** elderly patients, modified supine, nephrolithiasis, percutaneous nephrolithotomy

## Abstract

With increases in the aging population, conditions affecting older people and relevant surgical techniques are becoming more pertinent. Modified supine percutaneous nephrolithotomy (PCNL) is increasingly being adopted. There are limited data on the safety of this position in the elderly patient population. We describe our experience of the modified supine position in patients aged 70 years and older. Between April 2011 and March 2021, patients aged 70 years and older undergoing a modified supine PCNL performed by a single surgeon were prospectively evaluated. Data including patient age, operative time, complications, stone clearance, and length of stay were collected and analysed. Sixty-nine procedures were performed on 67 patients with a mean age was 76.5 years. Median total operative time was 95 min with 20 (29%) patients having a combined procedure with ureterorenoscopy. Preoperative mean stone burden was 23.5 mm and complete stone clearance was achieved in 46 (66.7%) patients. Twelve (17.4%) patients had complications during their hospitalisation. Six were Clavien–Dindo class II or less and one Clavien–Dindo class V. The modified supine position for PCNL is safe in the elderly patient population and has advantages including reduced handling of patients and achieving adequate stone-free rates. These benefits are particularly important in the elderly population, which frequently has a reduced tolerance to adaptation.

## 1. Introduction

Percutaneous nephrolithotomy (PCNL) is the treatment of choice for renal calculi larger than 20 mm or complicated stones such as staghorn calculi due to its superior efficacy in achieving stone clearance [1,2]. PCNL has evolved over time through the development of fluoroscopy and lithotripsy devices as well as variations to the technique [3]. However, PCNL is associated with some potential morbidity in comparison to other treatment modalities such as shockwave lithotripsy and ureterorenoscopy [4].

Continued improvements in life expectancy have seen the growth of the elderly population. According to the World Health Organization, it is anticipated that by 2050, there will be over two billion people aged 60 years and older [5]. With increased survival and the physiological changes of aging, the risk of developing multiple chronic diseases has increased. Thus, the growing elderly population has been reflected by an increase in the number of elderly patients with renal calculi. 

Untreated large stone burden in elderly patients must be given careful consideration. Deterioration of renal function should be prevented where possible to limit effects on coexisting medical conditions such as hypertension and diabetes mellitus [6]. There is also a risk of urinary tract infection and sepsis if the stones remain untreated. Both sepsis and renal failure pose the biggest risk of mortality from staghorn calculi [7]. The additional challenge of staghorn calculi is often the requirement of a second procedure to achieve acceptable results. 

Although age alone does not significantly influence operative risk, advanced age is associated with chronic diseases that contribute to an increased anaesthetic risk. Older patients have a generalised decline in organ function and a decreased adaptive reserve. These changes must be accounted for when selecting surgical candidates and the optimal technical approach for the elderly population [8]. 

The modified supine position has reported benefits including easier anaesthetic management, reduced operative time, and better access to the renal tract via anterograde or retrograde approach [9]. Furthermore, stone clearance rates are similar to prone positions. These benefits are advantageous in vulnerable populations such as the elderly. Although there have been studies on standard prone and supine PCNL techniques in the older patient demographic, since the introduction of the modified supine positioning, there is little literature describing outcomes in the elderly patient population of this alternate approach. The purpose of this report is to evaluate the safety and efficacy of the modified supine PCNL approach for the treatment of nephrolithiasis in patients aged 70 years and older. 

## 2. Materials and Methods

Patients who underwent modified supine PCNL at a metropolitan tertiary centre between June 2011 and March 2021 were prospectively recorded. The techniques used were previously described in our previous publication [10]. In summary, this technique is a modification to the described Galdakao-modified supine Valdivia position and involves patients lying on a C-shaped vacuum beanbag positioner on the operating table. The beanbag is further rotated so that a portion of it is draped below the operating table to allow a greater range of movements for the surgical instruments. When the patient is anaesthetised, their legs are placed in a lithotomy position with the ipsilateral hip flexed and the contralateral leg abducted and supported in an extended position. The beanbag is rolled under the hips to tilt the torso at an angle of approximately 20–30 degrees. The ipsilateral arm with a flexed elbow is placed on an arm support over the chest and the contralateral arm is tucked next to the toros with an extended elbow. Suction is applied to the beanbag, allowing it to support the tilted patient. If a combined technique is required, a second surgeon can simultaneously perform ureteroscopy through an access sheath.

The number and position of puncture were dependent on calculus size and location. Ureteric stents and nephrostomy were placed if clinically indicated. 

A sub-group analysis has been performed on patients older than 70 years of age. Information collected included sex, body mass index (BMI), comorbidities, stone burden and position, puncture site, peri-operative complications, length of patient stay, and stone clearance. Operative time was defined as total time spent in the operating room including positioning, anaesthetic, and surgical time. All patients had a preoperative urine culture and any infections were treated prior to surgery. 

The standard preoperative radiological study was a Computed Tomography (CT) KUB (kidney, ureter, and bladder). The stone burden was assessed based on maximum diameters on pre-operative imaging. Stone-free status and residual stone burden were defined according to the visualisation of any calculi on post-operative imaging. Post-operative imaging included X-ray KUB, CTKUB, or it was not documented. Patients were reviewed until the most recent follow-up. Complications were recorded and classified according to the Clavien–Dindo Classification [11]. Grading of the complexity of the PCNL was adapted according to Guy’s stone score [12]. 

Ordinal outcomes were compared using the Chi-Square test whilst continuous variables were compared using the Kruskal–Wallis test. IBM SPSS statistics (version 25, IBM Corp., Armonk, NY, USA) was used for statistical analysis and a *p*-value less than 0.05 was considered statistically significant.

The study was approved by the local Human Research Ethics Committee (Ethic Committee Name: Monash Health Human Research Ethics Committee; Approval Code: RES-21-0000-228Q—75328).

## 3. Results

A total of 69 procedures were performed on 67 patients (two patients had procedures on both sides). Patient demographics are shown in Table 1. The mean patient age was 76.5 years (range 70–89), with approximately two-thirds of patients (64.7%) being male. Half of the group had an American Society of Anesthesiologists (ASA) score of 2 or less and 44.1% had an ASA of 3. Mean operative time was 101.7 min and median length of inpatient stay was one day (range 0–26 days). The patient who had a 26-day admission was an outlier, as they were transferred from a secondary centre following a prolonged two-month admission for stone-related sepsis and required perioperative optimisation prior to their elective operation. 

Amongst the patients, the majority were either classed as overweight or obese, with a median BMI of 27.7 (range 18.8–39.1). Most of the patients had one or more comorbidities. The most common comorbidity was hypertension, affecting 56.7% of patients. Eighteen (26.9%) had type II diabetes and ten (14.9%) had ischaemic heart disease. 9% had chronic kidney disease and 9% had airway disease. Twenty-three patients (34.3%) were on antithrombotic agents—thirteen patients were on aspirin, three on clopidogrel, three on dual antiplatelet therapy, three on warfarin, and one on a new oral anticoagulant. All anticoagulants were stopped for the recommended periods prior to surgery.

The preoperative mean stone burden was 23.5 mm. Stone-free status was achieved in 47 (68.1%) patients. Twenty patients (29%) underwent a combined procedure with ureterorenoscopy because of stone complexity. A ureteric stent was placed in one-third of the patients. Half of the patients (50.7%) had a nephrostomy placed. Twenty procedures (29%) were tubeless (Table 2). Patients who had both nephrostomy and stent placements had a significantly longer hospital stay (*p* = 0.005). Complications were highest in the cohort that had both nephrostomy and stent (33.3%), followed by patients that had a stent placed (28.6%).

The median time to follow-up was 99 days. In the 22 patients who had residual stones, the mean residual stone burden was 11.2 mm (range 2–28 mm). The mean preoperative stone burden in patients with residual stones was 26.9 mm. Fourteen (20.3%) patients had a secondary procedure for residual stones. Among these patients, seven underwent a pyeloscopy, and one was scheduled to have a relook pyeloscopy. Three patients had a ureteroscopy, and one was scheduled for a relook ureteroscopy. Two patients had shockwave lithotripsy.

Post-procedure complications were observed in twelve (17.4%) patients as detailed in Table 3. Half were Clavien–Dindo class II or fewer complications. All three Clavien–Dindo IVa complications were patients who developed post-operative sepsis and required admission into the Intensive Care Unit. There was one death due to pulmonary emboli in a patient who had a prolonged pre-operative admission of two months. Two patients (2.9%) required a blood transfusion. One of them developed haematuria and a subcapsular renal haematoma and was on a blood-thinning agent for atrial fibrillation. The second patient had intraoperative blood loss of one unit of blood.

## 4. Discussion

The advantages of a modified supine position have been well documented. These can be divided into patient, anaesthetic, surgeon, and theatre staff factors. Patient positioning is particularly critical in the elderly population. Osteoporosis, decreased muscle tone, and arthritic conditions contribute to a higher risk of positioning complications such as skin breakdown, dislocations, and musculoskeletal pain. Therefore, eliminating the need to reposition for a prone position decreases the risk of such complications. There is also a reduced risk of skin pressure injuries and the rare serious complication of blindness associated with the prone position. In addition, elderly patients have twice the likelihood of experiencing cerebral desaturation in the prone position compared to supine [13]. Cerebral desaturation has been associated with postoperative cognitive dysfunction and longer hospital admissions. 

A meta-analysis by Liu demonstrated a significantly shorter operative time of supine PCNLs compared to prone positions [14]. Operative duration is an important consideration as longer times increase mortality risk in patients aged 80 years and over. Thirty-minute increments in operative time increase the odds of mortality by 17% in this patient group [15]. Compared to the studies with supine patients included in the meta-analysis, the operative time in our cohort was longer with a mean of 101.7 min. However, this included anaesthetic time and the caution and challenges of anaesthetic care in elderly patients may have contributed to our cohort’s extended theatre time.

Anaesthetic benefits of a modified supine position include easier access to the airways for the duration of the procedure, not having to reposition the patient, and avoiding the prone position [10]. The prone position can increase sympathetic activity, reduce venous return and potentially obstruct the inferior vena cava, requiring an increased depth of anaesthesia [16]. There can also be difficulties ensuring the correct placement of the endotracheal tube when turning the patient and increased ventilation requirements.

Benefits for the surgeon include reduced radiation exposure with the hands operating away from the fluoroscopic field, shorter operating times, and spontaneous expulsion of stones through the tract [17,18]. Two surgeons can also perform simultaneous retrograde and anterograde approaches to assist with access to large staghorn calculi and ureteric calculi [10]. This therefore provides additional benefits of potentially reducing operating time and improving stone clearance. From an ergonomic perspective, a modified supine approach allows the surgeon to be seated during prolonged parts of the procedure. The benefits for operating room personnel include a reduced workload, as there is no requirement to physically reposition the patient or re-drape the sterile field.

The overall complication rate in the study was 17.4%, which is within the range of 8–20.5% observed in modified supine groups in studies by De Sio et al. and Jones et al. [19,20]. However, there are limitations to these comparisons as complications were classified differently amongst the various studies. Furthermore, this single figure does not capture the rarity of major complications, such as the one death that was observed in our study. Alongside a generalised decline in organ function, elderly patients are less adaptive in particular to acute stresses that occur during the perioperative period [8]. The reported transfusion rate (2.9%) and significant sepsis rate (4.3%) were similar to previous studies [14,19].

In this cohort, the majority of complications were related to pre-existing co-morbidities, emphasising the role of preoperative evaluations to identify those at risk of postoperative complications. Furthermore, the role of multidisciplinary care involving perioperative physicians and geriatricians facilitates the comprehensive identification and management of postoperative complications related to advancing age [21]. The episodes of sepsis were related to infected calculi and despite pre-operative treatment with appropriate antibiotics; a septic shower was likely caused by the breaking of calculi and release of bacteria during the urological procedure. The one case of left kidney pseudoaneurysm may be the only complication directly related to the technique. However, renal artery pseudoaneurysms are an associated iatrogenic injury of PCNL (and other renal procedures) with an incidence of 1–3% [22]. The patient made a full recovery following successful coil embolisation of the pseudoaneurysm.

The mean length of hospital stay of 2 days in this study was similar to other studies in the meta-analysis by Liu et al. [14]. Prolonged hospital stays are a predictor of functional decline in older patients, with increased risk of hospital-acquired complications such as nosocomial infections and increased health-care costs. 

In our study, 50.7% of patients had a nephrostomy placed. This is a lower rate compared to Okeke et al. who noted 93.4% of elderly patients undergoing PCNL had a nephrostomy [23]. In 2015, Ozturk et al. reported that tubeless PCNL is a safe method for the elderly population [24]. Its advantages are the reduction of analgesic requirements, shortened length of stay, allowing for early mobilisation and a decrease in complications. However, a nephrostomy tube allows renal drainage and the possibility of tamponade bleeding from renal access. Thus, selection of nephrostomy tube placement by the surgeon must factor in several elements such as postoperative care, length of stay, and potential for re-access or bleeding. It is reassuring that there were no complications reported in the tubeless group. This confirms that in selected patients a tubeless approach is appropriate and safe. Stent placement rates between our study and Okeke et al. were similar (33.3% and 39% respectively); however, there are limited data on the effects and safety of stent placement in PCNL in the geriatric population. Virtually all the stents in this study were related to the more complex cases involving a second surgeon performing simultaneous ureterorenoscopy.

Stone-free status was achieved in 68.1% of patients in our study, which is similar to the stone-free rates produced in other studies where PCNL was performed in elderly patients, ranging from 36–78.3% [25,26,27]. This suggests that the use of the modified supine position does not impact stone clearance rates. However, clearance rates in our study did not include re-treatment outcomes, as they were performed by other surgeons. Furthermore, stone-free rates were defined differently among the studies. Some studies suggest that “near-stone” free status in elderly patients can be acceptable as long-term complications of minimal residual stones are unlikely to occur [28]. Although residual stones can serve as a nidus for recurrent urinary tract infections and propagate further fragments, aggressive stone clearance must be carefully considered in the comorbid and frail elderly patient.

Our stone-free results, complication, and length of stay rates appear similar to other studies. However, this is not a comparative study, as highlighted, there are variabilities in reporting of evaluation of renal stone treatments [29]. There are therefore caveats to making a fair comparison. Other limitations of this study include that it is retrospective and a single surgeon series with no comparison group. 

This study supports the safety of modified supine PCNL in elderly patients. Although elderly populations may confer higher anaesthetic risks, the advances of PCNL with a modified supine approach allow for stone clearance in a short time whilst minimising complications. There are advantages such as reduced handling of patients and benefits from an anaesthetic and theatre staff perspective. Along with careful pre-operative patient assessments, proper case selection, and good anaesthetic management, this procedural technique can be safely used to yield better patient outcomes for the treatment of significant stone disease. 

## Figures and Tables

**Table 1:** Patient and operative characteristics.

	Value
Age	
Age (y.o), mean (sd)	76.5 (4.1)
Age range (min–max)	70–89
Gender	
Male (%)	64.7
Female (%)	35.3
Body Mass Index	
BMI, mean (sd)	28 (4.7)
BMI range (min–max)	18.8–39.1
ASA Classification	
1 (%)	4.5
2 (%)	48.5
3 (%)	44.1
4 (%)	2.9
Stone Burden	
Stone size (mm), mean (sd)	23.5 (10.4)
Stone size range (mm), (min–max)	10–60
Patients with multiple stones (%)	32.4
Side	
Left (%)	43.5
Right (%)	56.5
Renal Puncture Site	
Lowe Pole (%)	61.8
Middle Pole (%)	29.4
Upper Pole (%)	8.8
Procedural Characteristics	
Operative duration (mins), mean (sd)	101.7 (42.7)
Operative duration (mins), median (range)	95 (30–260)
Length of hospital stay (days), mean	2
Length of hospital stay (days), median (range)	1 (0–26)
Patients requiring blood transfusion (%)	2.9
Residual stones (%)	31.9
Size of residual stones, mean (mm)	11.2
Follow-up treatment (%)	20.3
Surgical Complexity	
1 (%)	64.7
2 (%)	10.3
3 (%)	14.7
4 (%)	10.3

y.o—years old; sd—standard deviation.

**Table 2:** Postoperative stent and nephrostomy placement.

	Only Stent Placed	Only Nephrostomy Placed	Stent and Nephrostomy Placed	No Stent or Nephrostomy	*p* Value
Number of patients (%)	14 (20.3)	26 (37.7)	9 (13)	20 (29)	
Number of complications (%)	4 (28.6)	5 (19.2)	3 (33.3)	0 (0)	0.069
Length of stay (days), median (range)	1 (0–2)	1 (1–26)	2 (1–6)	1 (1–2)	0.005

**Table 3:** Postoperative complications according to Clavien–Dindo classification.

Clavien–Dindo Classification/Complication	No. of Patients
**Grade I**	
Supraventricular tachycardia	1
Pulmonary oedema	1
**Grade II**	
Fevers	1
Hypotension	1
Hypoxia and tachycardia	1
Cellulitis	1
**Grade IIIa**	
Left kidney pseudoaneurysm	1
**Grade IIIb**	
Haematuria and subcapsular renal haematoma	1
**Grade IVa**	
Sepsis	3
**Grade V**	
Death	1

## Data Availability

The data presented in this study are available upon reasonable request from the corresponding author.
